# Reflections on End-of-Life Dialysis

**DOI:** 10.1590/2175-8239-JBN-2018-00030003

**Published:** 2018-09-03

**Authors:** Carmen Tzanno-Martins

**Affiliations:** 1 Sociedade Brasileira de Nefrologia, São Paulo, SP, Brasil.; 2 Sociedade Latinoamericana de Nefrologia e Hipertensão, São Paulo, SP, Brasil.

Chronic kidney disease (CKD) usually progresses more slowly in elderly patients. Therefore, they may stay for longer periods of time on conservative management.

Life expectancy and quality of life tend to deteriorate in elderly individuals with pre-dialysis CKD and older patients on dialysis. Furthermore, there is controversy over the benefits of dialysis and when to start or stop it.

The first three months of dialysis are critical, with patients experiencing decreases in functional capacity and quality of life.^1^ Life expectancy decreases, as 69% of the patients aged 75 years or older survive after one year of dialysis and a meager 20% are alive after five years of treatment.^2^ Individuals aged 90 years or older are expected to survive for eight months.^3^

The annual census survey of hemodialysis centers organized by the Brazilian Society of Nephrology revealed an increase in the number of individuals aged 65 years or older on dialysis. In the last decade this segment of the patient population increased by 42%, and today approximately 30% of the individuals on dialysis are elderly.^4^

Elderly patients on dialysis suffer from increased risk of falls^5^ and present more clinical comorbidities and bouts of severe anemia.

Most elderly on hemodialysis are fragile - a condition associated with worse clinical outcomes and mortality - and partially or completely dependent on family members or caregivers.

Old age is also a risk factor for cognitive impairment, which in turn increases mortality by 1.5 to 2 times.^6^

Blood pressure oscillations during dialysis, rapid volume changes, vascular access issues, and electrolyte imbalances aggravate cognitive deficit.

A recent comparative study revealed that dialysis does not improve the survival of individuals aged 80 years or older.^2^ Conservative management has been associated with higher levels of patient satisfaction, better quality of life, and mean survival of 13 months.^7^

In this issue, the author of "Reflections on Endof-Life Dialysis" covered issues seldom addressed in nephrology services: decision-making; shared decision-making; the tools we have; the vulnerability of physicians; healthcare worker and aregiver burnout.^8^

Although healthcare professionals are aware that offering palliative care and early integration of this approach result in improved symptom management and better quality of life without reducing survival, most nephrologists still do not consider this option.

According to the author, who endorsed Kee's study, nephrologists differ on whom to offer dialysis; and what weighs most on this decision is the patient's mental state 9.

The decision to start or discontinue dialysis should be shared and made with the support of assessment instruments. Evaluating disease progression, mortality scores, and patient functional capacity and mental state increases team confidence.

The thought explored by the author in his paper that factors beyond clinical and psychical conditions ought to be considered - including cultural, religious, spiritual, educational, and legal aspects - resonated well with me.

An Internet survey recently organized by the Italian Society of Nephrology found that although nephrologists consider dialysis and chronic kidney disease to be elements inherent to aging, they are unaware of tools that might be used in patient assessment.^10^

Geriatricians look at indicators of fragility, functional disability, and risk of unfavorable outcomes. However, nephrologists are not used to resorting to such references and formulas, and seldom use them in their decisions.

The European Best Practice Group recently published a manual with clinical guidelines for elderly patients with CKD stage 3b or more severe, along with scores for clinical management and shared decision support^11^.

The BANSAL score^11^ is useful for predicting the 5-year risk of death of non-fragile elderly patients with CKD stages III to V.

The REIN^11^ (Renal Epidemiology and Information Network) score is useful in the stratification of risk of death of frail elderly patients considered for dialysis.

The Kidney Failure Risk Equation (KFRE)^10^ has excellent predictive accuracy of risk of CKD progression.

Considering disease progression, mortality risk, comorbidities, and patient fragility, nephrologists could make shared decisions focusing on renal protection and renal support actions while planning for advanced care and/or pre-dialysis counseling to discuss the choice between modes of dialysis and conservative management.^11^

Decisions are often complex and entail a high degree of uncertainty, even when scores and scales are used to aid decision-making and the sharing of responsibility.

A commonly neglected issue that reflects on team performance is burnout, as mentioned by the author. Patients with longstanding chronic diseases and elderly individuals require greater attention from multiprofessional teams and caregivers. Older patients often suffer with loss of independence, absenteeism at work, poor socialization, impoverishment due to reduced income, and increasing expenses with healthcare and family restructuring. Therefore, a trained multiprofessional team and efficient communication and technologies are required to treat these patients.

In 2015, the KDIGO published an executive summary on renal support in CKD on account of the growing population of elderly individuals on dialysis, in an attempt to tackle questions over which might be the best treatments yielding greater benefits.

In addition to the factors considered by the author in his paper, the following might also be deemed as recommendations to improve shared decision-making and the communication between the team, their patients, and caregivers:

1. Develop a doctor-patient relationship in which decision-making is an integral element.
2. Inform patients with AKI and CKD stages 4 and 5 of their diagnoses, prognoses and treatment options.
3. Provide patients with AKI and CKD stages 4 and 5 with a prognosis for their general condition.
4. Report the care for both renal replacement therapy and renal support.
5. Avoid starting or discontinuing dialysis when patients and/or their families express a desire for renal support care.
6. Consider discontinuing or not starting dialysis for patients with AKI and CKD with poor prognoses including end-stage renal disease, medical conditions that hinder dialysis and patients with two or more of the following:
  - Age of 75 years and older.
  - High comorbidities index (Charlson > 8).
  - Functional disability (Karnofsky or PPS < 40).
  - Chronic malnutrition.
7. Consider offering dialysis for a limited time for patients with uncertain prognoses or unclear benefits from undergoing dialysis.
8. Establish a conflict resolution plan for shared decisions.
9. Provide support to manage the symptoms and complications of patients on renal support.
10. Communicate the decisions to all staff involved.

Life expectancy is on the rise and the number of elderly with chronic diseases tends to increase. Therefore, the reflections proposed by the author are relevant and current. Nephrologists must consider palliative care as a therapeutic option, and find tools to make decisions and share the ensuing challenges with other professionals, caregivers and the patients themselves.

## References

[1] Verberne WR, Geers AB, Jellema WT, Vincent HH, van Delden JJ, Bos WJ (2016). Comparative survival among Older Adults with Advanced Kidney
Disease Managed Conservatively Versus with Dialysis. Clin J Am Soc Nephrol.

[2] Murtagh FE, Marsh JE, Donohoe P, Ekbal NJ, Sheerin NS, Harris FE (2007). Dialysis or not? A comparative survival study of patients over 75
years with chronic kidney disease stage 5. Nephrol Dial Transplant.

[3] Kurella M, Covinsky KE, Collins AJ, Chertow GM (2007). Octagenarians and nonagenarians starting dialysis in the United
States. Ann Intern Med.

[4] Sesso R, Lopes AA, Thomé FS, Lugon J, Tzanno-Martins C (2017). Brazilian Chronic Dialysis Survey 2016. J Bras Nefrol.

[5] Plantinga LC, Patzer RE, Franch HA, Bowling CB (2017). Serious Fall Injuries Before and After Initiation of Hemodialysis
Among Older ESRD patients in the United States: A Retrospective Cohort
Study. Am J Kidney Dis.

[6] Kurella M, Mapes DL, Port FK, Chertow GM (2006). Correlates and outcomes of dementia among dialysis patients: the
Dialysis Outcomes and Practice Patterns Study. Nephrol Dial Transplant.

[7] Carson RC, Juszczak M, Davenport A, Burns A (2009). Is maximum conservative management an equivalent treatment option
to dialysis for elderly patients with significant comorbid
disease?. Clin J Am Soc Nephrol.

[8] Castro MCM (2018). Reflections on end-of-life dialysis. J Bras Nefrol.

[9] Kee F, Patterson CC, Wilson EA, McConnell JM, Wheeler SM, Watson JD (2000). Stewardship or clinical freedom? Variations in dialysis decision
making. Nephrol Dial Transplant.

[10] Antonelli Incalzi R, Aucella F, Leosco D, Brunori G, Dalmartello M, Paolisso G (2015). Assessing Nephrological Competence among Geriatricians: A Proof
of Concept Internet Survey. PLoS One.

[11] Farrington K, Covic A, Aucella F, Clyne N, de Vos L, Findlay A, ERBP Guideline Development Group (2016). Clinical Practice Guideline on management of older patients with
chronic kidney disease stage 3b or higher (eGFR <45 mL/min/1.73
m2). Nephrol Dial Transplant.

